# Surface Plasmon Resonance Based Binding Characterization for Screening RNA-Loaded Lipid Nanoparticles (LNPs): Exploring Species Cross-Reactivity in LNP–Apolipoprotein E Interactions

**DOI:** 10.1021/acs.molpharmaceut.5c00068

**Published:** 2025-07-07

**Authors:** Benjamin Lew, Sandeep Chhabra, Jacob A. Lewis, Angela C. Wagoner, Angel Hsu, Steve Halaby, Pooja Sharma, Justin K. Murray, Francis Kinderman, Deirdre Murphy Piedmonte, Brendan R. Amer

**Affiliations:** † Pre-Pivotal Drug Product Technologies, Process Development, Operations, Amgen, Thousand Oaks, California 91320, United States; ‡ Amgen Postdoctoral Fellow Program, Amgen, Thousand Oaks, California 91320, United States; § Lead Discovery and Characterization, Research, Amgen, Thousand Oaks, California 91320, United States; ∥ Complex Biologics, Amgen Research, Amgen, Thousand Oaks, California 91320, United States

**Keywords:** Lipid nanoparticles, Surface plasmon resonance, Apolipoprotein E, Species cross-reactivity, Formulation screening

## Abstract

Lipid nanoparticles (LNPs) are an essential delivery platform for nucleic acid payloads that are susceptible to degradation or elimination. Upon administration, a biomolecular corona composed of serum proteins forms around the LNP surface, which is crucial for tissue targeting and biodistribution. One essential protein that drives the distribution of LNPs is apolipoprotein E (ApoE). In this work, we used ApoE as a model protein to probe LNP–protein interactions utilizing a surface plasmon resonance (SPR) interaction assay comparable to the conventional quartz crystal microbalance with dissipation (QCM-D) assay. We employed two LNP formulations with or without payload and various ApoE homologs to establish the SPR method as an *in vitro* tool to screen LNP formulations and explore species cross-reactivity in LNP–protein interactions. Two binding models were applied to capture the difference in binding behaviors of different ApoE homologs in terms of relative binding response and association kinetics.

## Graphical abstract

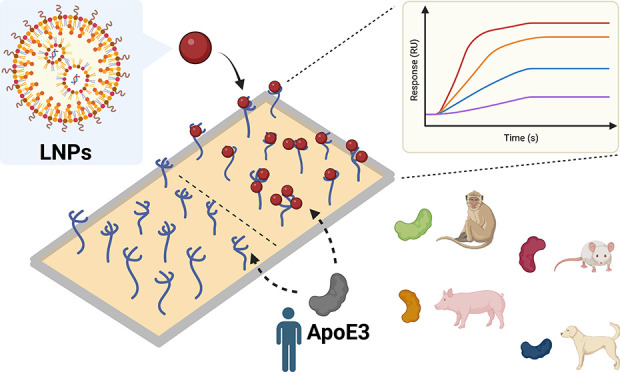

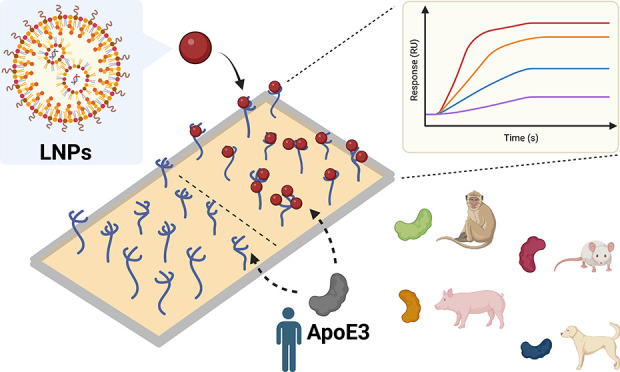

## 1 Introduction

Lipid nanoparticles (LNPs) are a promising platform for delivering nucleic acid molecules to treat and prevent complex diseases, including cancer, genetic disorders, and infectious diseases.^1,2^ Nucleic acids such as small interfering RNAs (siRNAs) and messenger RNAs (mRNAs) achieve therapeutic effects through gene silencing and gene expression, respectively.^3,4^ However, there are major challenges limiting the effective delivery of these nucleic acids, including their susceptibility to degradation and poor stability *in vivo*. Hence, LNPs can be employed as protective carriers to safely deliver nucleic acids by encapsulating the payload and improving their cellular uptake, thereby enabling nucleic acids to regulate the expression of the target gene of interest.^5,6^

The *in vivo* biodistribution and target-specificity of LNPs are crucial factors to understand because they determine the efficacy of LNP-mediated gene therapy. LNPs are predominantly comprised of five components: ionizable cationic lipid, helper lipid, polyethylene glycol (PEG)-lipid, cholesterol, and the nucleic acid payload. Varying the components and their ratios alters the physicochemical properties of the formulated LNPs. Upon *in vivo* administration and subsequent biodistribution, LNPs encounter a variety of serum proteins, leading to the formation of a biomolecular corona on the LNP surface. Among them, apolipoprotein E (ApoE) is the most abundant serum protein forming the corona, thus playing a pivotal role in the pharmacokinetics of LNPs.^7^ For instance, patisiran (Onpattro), the first FDA-approved LNP-siRNA, targets hepatocytes through an ApoE-mediated low-density lipoprotein receptor (LDLR) pathway.^8,9^ Because ApoE is the essential arbiter determining the fate of the LNP-mediated delivery, there is a need for a binding assay capable of quickly testing the affinity of different LNP formulations for ApoE and other serum or target proteins.

Given the distinct physicochemical characteristics and heterogeneity of RNA-loaded LNPs, it is essential to establish a comprehensive analytical procedure to evaluate these complex formulations.^10,11^ Currently, LNP characterization is commonly done through *in vitro* and *in vivo* assays. One such *in vitro* assay includes surface-interaction assay techniques like quartz crystal microbalance with dissipation (QCM-D), which can screen different LNP formulations by assessing their binding affinity with target macromolecules.^12,13^ While QCM-D is a powerful technique and the current standard in the field, it typically requires a large sample volume, complicated preparation steps, and is generally low-throughput.^14^

Here, we present a highly sensitive and time-efficient screening assay utilizing surface plasmon resonance (SPR) to characterize LNP formulations by assessing their binding kinetics with proteins of interest. SPR is a versatile technique that can investigate the surface functionalization of nanoparticles and their interaction with biomolecules at low volumes and concentrations.^14^ PEG-specific antibody-functionalized sensors enable the association of LNPs on the SPR surface to monitor the interaction between LNPs and specific target proteins by measuring the shift in resonance angle in SPR.

We tested two LNP formulations with different ionizable lipids to demonstrate the binding interaction between LNPs and ApoE. The relative binding response, kinetics, and affinities were assessed and compared among different proteins using our SPR assay format. Additionally, we investigated the species cross-reactivity of ApoE-LNP interactions for five different ApoE homologs not only to demonstrate the versatility of this binding assay but also to establish a robust tool to inform on the validity of non-human toxicology or pharmacokinetic (PK) studies for LNP-based therapeutics.

## 2 Materials and Methods

### 2.1 Materials

DLin-MC3-DMA (MC3), SM-102, 1,2-Distearoyl-*sn*-glycero-3-PC (1,2-DSPC), DMG-PEG(2000), and cholesterol were purchased from Cayman Chemicals (Ann Arbor, MI, USA). CleanCapFirefly Luciferase mRNA (5-methoxyuridine) was purchased from TriLink BioTechnologies (San Diego, CA, USA). siRNA was synthesized in house.^15^ Human ApoE3 protein, mouse ApoE protein, and cynomolgus ApoE were purchased from Acro Biosystems (Newark, DE, USA). Pig ApoE and Dog ApoE were purchased from Creative Biomart (Shirley, NY, USA).

A biotin anti-PEG antibody (PEG-B-47b) was purchased from Abcam (Cambridge, UK). N-Biotinyl-3,6-dioxaoctane-1,8-diamine (Biotin-(PEO)­3-amine) was purchased from Neta Scientific (Marlton, NJ, USA). Biotin CAPture Kit, Series S and HBS (HEPES buffer saline: 0.01 M HEPES, 0.15 M NaCl, pH 7.4) buffer were purchased from Cytiva (Marlborough, MA, USA). Bovine serum albumin (BSA) and human serum albumin (HSA) were purchased from Sigma-Aldrich (Saint Louis, MO, USA). Biotin functionalized QCM-D sensors (QSX 339) were purchased from Biolin Scientific (Gothenburg, Sweden).

### 2.2 LNP Formulation and Characterization

Two ionizable lipids, MC3 and SM-102, were selected to prepare two cationic LNP formulations for this study. LNPs were formulated using a NanoAssemblr Benchtop microfluidic instrument (Precision Nanosystems, Vancouver, BC, Canada). The stock solution of each lipid component was prepared in ethanol and mixed at the molar ratio of 50:10:1.5:38.5 mol % (cationic lipid:DSPC:DMG-PEG:cholesterol). The mRNA and siRNA solutions were prepared in sodium acetate buffer (pH 4.0).

Initially, empty LNPs were formulated by loading the NanoAssemblr with 1 mL lipid mixtures and 3 mL of sodium acetate buffer at 12 mL/min. Subsequently, RNA-loaded LNPs (RNA-LNPs) were formulated by feeding the instrument with 1 mL lipid mixtures and 3 mL of designated RNA solution with an N/P ratio of 6:1 at 12 mL/min. The resulting LNPs and RNA-LNPs were buffer exchanged into an SPR running buffer (HBS). The resulting LNPs and RNA-LNPs were characterized by assessing their hydrodynamic diameter and zeta-potential (Zetasizer Nano, Malvern Panalytical, Malvern, UK) and nanoparticle concentrations (NanoSight NS500, Malvern Panalytical). The RNA encapsulation efficiency was assessed with the Quant-it RiboGreen RNA Assay Kit (ThermoFisher Scientific, Waltham, MA) and flow nanoanalyzer (NanoFCM, Nottingham, UK).

### 2.3 SPR Sensor Functionalization

Biacore Series S Sensor Chip CAP was inserted into the SPR instrument (Biacore T200, Cytiva) and conditioned with three 1 min injections of regeneration solution (6 M guanidine-HCl + 0.5 M NaOH) and rinsed with running buffer (HBS). Then, the three channels in the sensor were functionalized with biotin anti-PEG antibody (aPEG, 6 μg/mL) at 5 μL/min for 300 s, followed by biotin-(PEO)­3-amine (50 mM) at 5 μL/min for 60 s. After rinsing the sensor with running buffer, the LNP solution (1 × 10^9^ particles/mL in HBS) was injected at 10 μL/min for 300 s. The reference channel was functionalized solely with biotin-(PEO)­3-amine to prevent the nonspecific binding of proteins.

### 2.4 LNP–ApoE Interaction Assessment

HSA and ApoE samples were prepared in a running buffer in a serial dilution of 300, 100, 33, 11, and 0 nM (for background subtraction). Once the sensor was functionalized, each sample was fed to the sensor at 30 μL/min for 300 s (association phase). Then, the sensor was rinsed with a running buffer for 500 s (dissociation phase). At the end of each cycle, the sensor was treated twice with a regeneration solution at 30 μL/min for 60 s to regenerate the sensor surface by removing all the bound molecules (aPEGs, LNPs, and proteins). A sensor channel without the bound LNPs was used as a reference and any response coming from it was subtracted from the LNP-channels to acquire active responses. Each response was normalized by the average capture level of LNPs (1,000 RU). The acquired sensorgrams were evaluated with the Biacore Insight Evaluation software (Cytiva) with either the 1:1 binding model or the two-step binding model to assess the kinetics and concentration response of each sample.

### 2.5 QCM-D Assessment

Biotin-functionalized QCM-D sensors (QSX 339) were mounted in the QCM-D flow modules (QSense Explorer, Biolin Scientific) and rinsed with PBS at 100 μL/min and 25 °C for an hour to acquire stabilized harmonic signals prior to the experiment. To prepare the sensors for LNP association, initially, each was treated with 1 mL of streptavidin (0.4 μM in PBS) followed by 1 mL of aPEG solution (1.5 μM in a running buffer) at 100 μL/min. Subsequently, the sensors were rinsed with a running buffer for 10 min to remove unbound ligands. Afterward, 1 mL of LNP solution (1 × 10^8^ – 1 × 10^10^ particles/mL in a running buffer) was fed to the flow module at 100 μL/min. Once the harmonic signals were stabilized after buffer wash, BSA, HSA and ApoE samples prepared in three concentrations (0.3, 1.5, and 3.0 μM) were fed to the flow module to assess the binding interaction between the samples and the bound LNPs. After the assessment, the sensors were regenerated by treating them with a regeneration solution containing 0.5% mol CHAPS detergent and 0.1% mol Triton-X which removed bound LNPs and proteins. The change in sensor thickness upon each LNP-sample interaction was assessed using the QSense Dfind software (Biolin Scientific, Gothenburg, Sweden), where the viscoelastic model and Sauerbrey model were applied to four harmonic overtones (*n* = 3, 5, 7 and 9).

### 2.6 Statistical Analysis

Data points in triplicate or more were expressed as mean values ± standard deviation and one-way ANOVA with Tukey post hoc test was performed to indicate the statistical significance of each data set. The difference between groups was considered significant at *p* < 0.05.

## 3 Results and Discussion

### 3.1 Hydrodynamic Diameter, Loading Efficiency, and Concentration of LNPs

Two LNP formulations were evaluated for size, loading efficiency and concentration. DLin-MC3-DMA-LNPs (MC3-LNPs) and SM102-LNPs were prepared to incorporate siRNA and mRNA, respectively (Figure 1a). The concentrations of the lipid components were constant for each formulation. Figure 1b shows the average hydrodynamic diameters of the two formulations before and after incorporating the RNA cargoes, which align with the average sizes reported in the literature.^16,17^ The average diameter of empty MC3-LNPs was 59.70 ± 5.27 nm, and the size increased to 90.98 ± 8.10 nm after incorporating siRNAs with the N/P ratio of 6, which corresponds to the ratio between the amine group (N) of the ionizable lipid and the phosphate group (P) of the nucleic acid cargo. The average diameter of SM102-LNPs before and after incorporating mRNAs was 73.40 ± 5.20 nm and 102.12 ± 7.28 nm, respectively. Empty SM102-LNPs were significantly larger than empty MC3-LNPs (*p* < 0.05), however, the difference between the two RNA-loaded LNPs was not significant (*p* = 0.07). ∼100 nm is the intended diameter of RNA-loaded LNPs for receptor-mediated endocytosis.^18,19^ The polydispersity index (PDI) of the empty and RNA-loaded LNPs was below 0.2, which indicates their relative homogeneity.^20,21^

**Figure 1:**
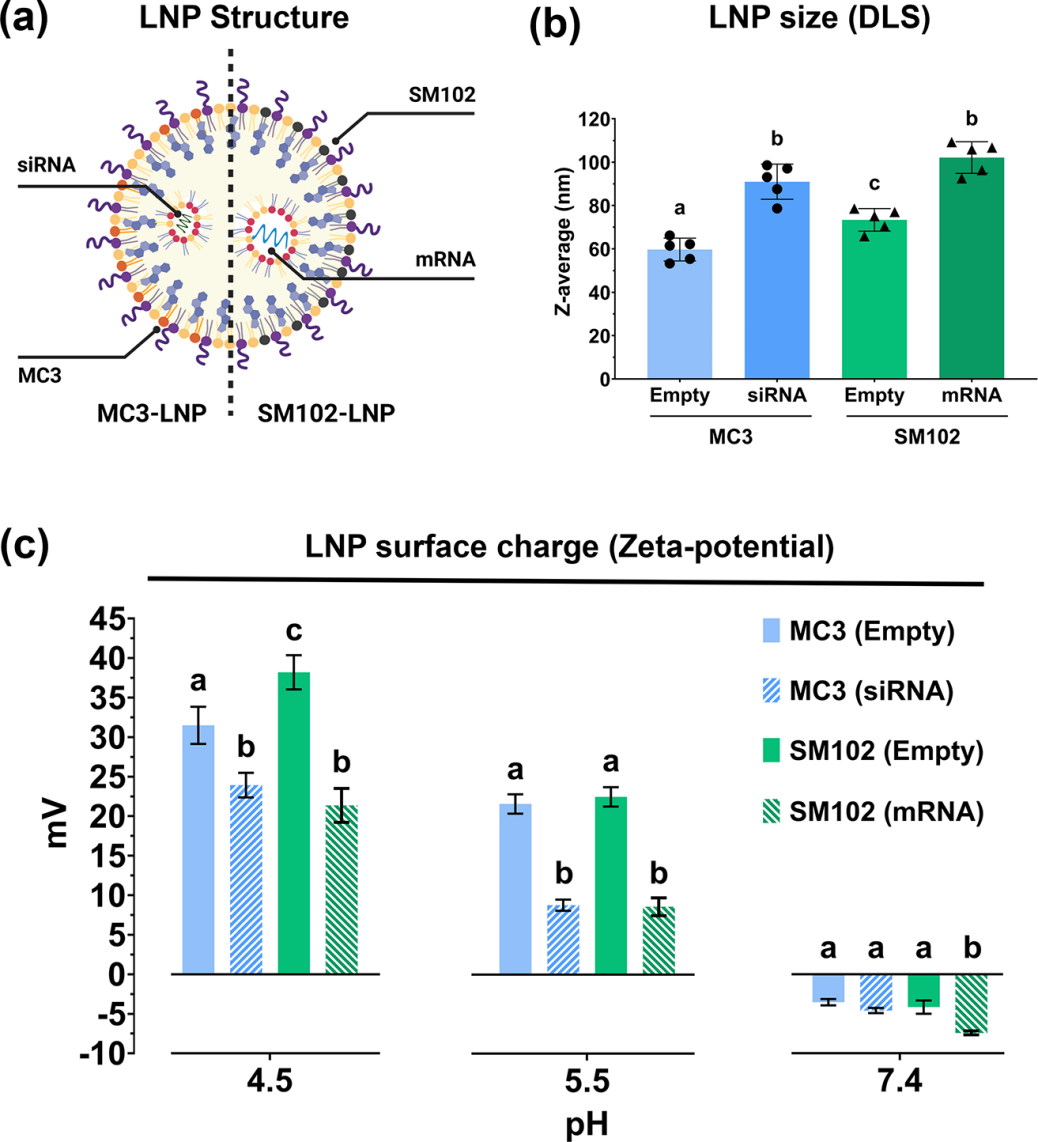
Formulation and characterization of LNPs. (a) Schematic illustration depicting the structural components of MC3- and SM102-LNPs encapsulating siRNA and mRNA, respectively. (b) Average hydrodynamic diameter of the two LNP formulations before and after encapsulating RNA cargoes (*n* = 5). Each letter denotes that the difference between groups is statistically significant (*p* < 0.05). (c) Zeta-potential measurements of the two LNP formulations before and after encapsulating RNA cargoes at three different pH (*n* = 3). At each pH, different letters denote that the difference between groups is statistically significant (*p* < 0.05).

The RNA loading efficiency of each batch was >80% when assessed with the RiboGreen assay, aligning with the previously reported results in the literature.^22,23^ We also observed that the majority (>90%) of the incorporated RNA cargoes were intact inside the LNP matrix, as shown by the flow nanoanalyzer scatter plots (Supplementary Figure 1). This ensures that no RNA cargoes will interfere with the LNP-protein interaction.

To determine LNP concentration, we used the nanoparticle tracking analysis (NTA) method to quantify the LNPs in terms of particles/mL. The average particle concentration of each LNP batch ranged from 2 to 5 × 10^9^ particles/mL after buffer exchange. The final concentration of all samples was adjusted to 1 × 10^9^ particles/mL with a running buffer before further assessment.

### 3.2 Zeta-Potential Assessment

The p*K*_a_ of the incorporated ionizable lipid determines the encapsulation and delivery efficiency of given LNP formulations, playing a central role in the biodistribution and potency of LNP-mediated RNA delivery.^24,25^ Once an LNP enters a cell via the endosomes, the pH drops to near 4.5, where the protonated LNP surface facilitates ion pair formation with anionic endosomal phospholipids to disrupt the endosomal membrane and release the payload within the cell.^24,26^ Hence, zeta-potential, which reflects the net surface charge of LNPs, is an essential parameter in understanding the stability and effectiveness of LNPs.^27^

The zeta-potential analysis in Figure 1c shows that the two LNP formulations, both empty and RNA-loaded, exhibited slightly negative surface charge at neutral pH. When the LNPs were exposed to an acidic environment (pH 5.5), they instead exhibited net positive charge with increasing magnitude as they transitioned to a more acidic environment (pH 4.5). Empty SM102-LNPs have slightly higher zeta-potential than empty MC3-LNPs in the acidic environment, and the difference was significant at pH 4.5 (*p* < 0.05). This is because the p*K*_a_ of SM-102 is 6.68, which is slightly higher than that of DLin-MC3-DMA (p*K*_a_ 6.44).^28^ The RNA-loaded LNPs exhibited lower zeta-potential magnitudes than the empty LNPs, which is another way to observe the incorporation of negatively charged RNAs in the LNP matrix. There was no noticeable difference between siRNA-loaded and mRNA-loaded LNPs with the same N/P ratio, except at pH 7.4 where mRNA-loaded LNPs exhibited more negative charge than the rest (*p* < 0.05). Still, all the formulations were considered neutral at pH 7.4 since the zeta-potential measurements were below −10 mV.^29^

### 3.3 Sensor Functionalization

Figure 2a illustrates the association of LNPs on the sensor surface through biotinylated anti-PEG antibodies (aPEGs) for the assessment of different proteins. The SPR assessment routine comprised sensor functionalization, LNP association, binding assessment, and sensor regeneration. aPEGs bind to DMG-PEG to capture the LNPs onto the sensor surface. After the association period, the signal was stable and no dissociation of LNPs was observed during the buffer wash step. Varying the exposure time of the aPEG solution to the sensor determined the response level to aPEG, and the aPEG response was in turn correlated with the response level to the LNPs following subsequent exposure of the LNP solution (Figure 2b). For kinetic analysis, about 1,000 Response Units (RU) were captured at each cycle of LNP association.

**Figure 2:**
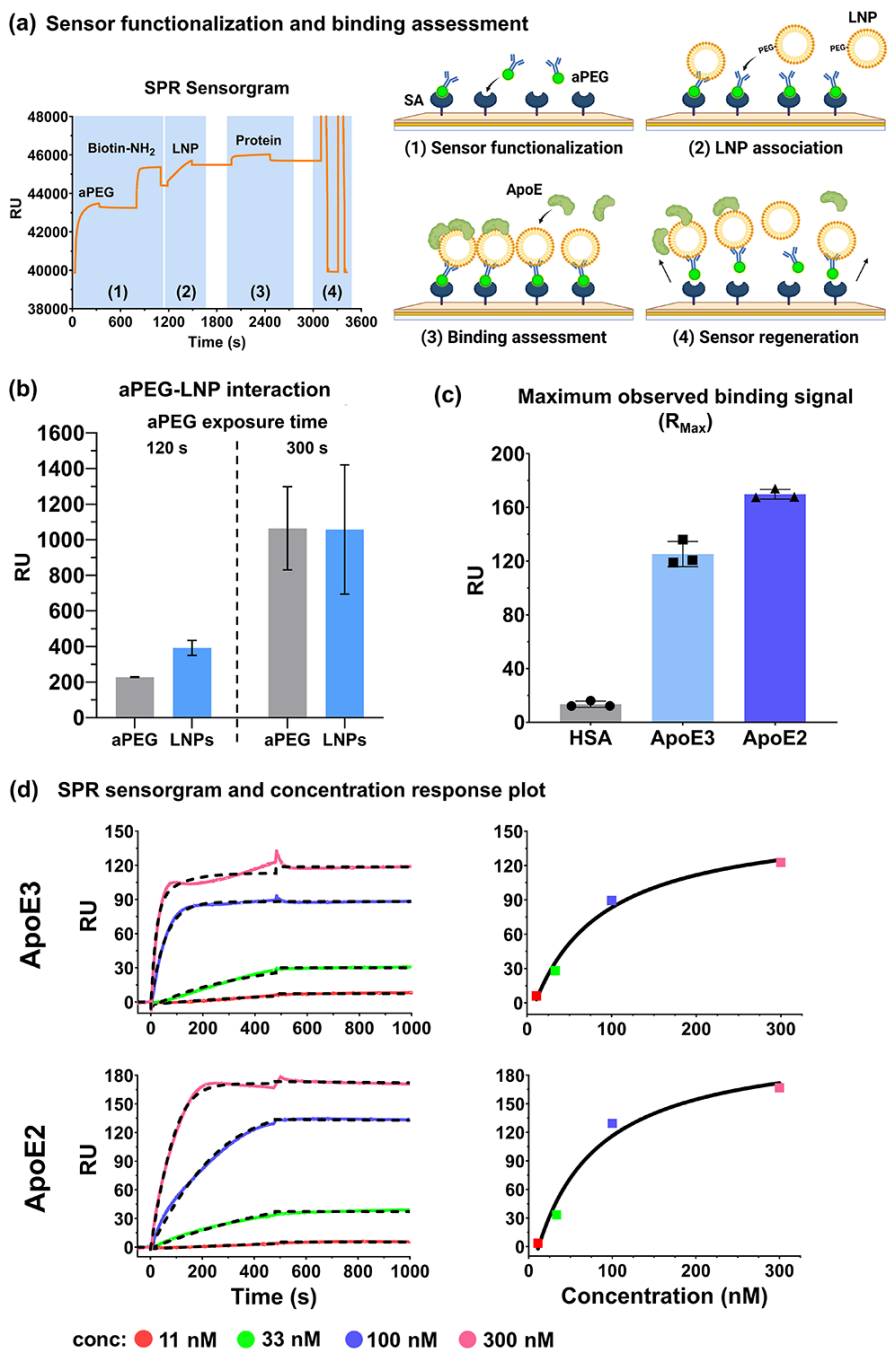
LNP interaction assessment via SPR. (a) Representative SPR sensorgram and schematic illustration depicting the sensor functionalization, LNP association, protein binding assessment and regeneration routine. (b) Average relative response level of the aPEG at two different exposure times (120 s, *n* = 6, and 300 s, *n* = 30, at 5 μL/min) and corresponding response from the LNP association (300 s at 5 μL/min). (c) Maximum observed binding signal (*R*_Max_) of empty MC3-LNPs with HSA, ApoE3, and ApoE2. (d) Representative sensorgrams and concentration–response plots of empty MC3-LNPs upon interacting with ApoE3 and ApoE2 in varying concentrations (11–300 nM).

In surface interaction assays, residual analytes from previous cycles may create false positive signals in subsequent cycles, particularly for those with high binding affinity to the ligand.^30^ Since serum proteins have strong interaction with LNPs to form stable biomolecular coronas, it is often difficult to separate them completely for the next cycle during the assessment. Hence, our method implemented a regeneration routine that basically eliminates all the associated molecules from the sensor surface to prevent any potential carryover. The SPR sensorgram in Figure 2a demonstrated the return of the signal to the baseline after the regeneration step, indicating the complete removal of aPEGs, LNPs and serum proteins from the sensor surface before the next cycle.

Subsequently, we utilized the QCM-D assessment as an independent method to validate the findings from the SPR assessment. The QCM-D sensors were functionalized with aPEGs as described previously.^13^ Unlike SPR, QCM-D assesses the change in mass on the sensor surface through a vibrating crystal that resonates at multiple harmonics comprising the fundamental (*n* = 1) and a set of overtones (*n* = 3, 5, 7···) with higher frequencies. Each overtone measures the changes in frequency and dissipation of energy upon molecule-sensor interaction. The aPEG functionalization achieved an average frequency and dissipation shift of −30 Hz and 0.6 ppm, respectively, and the sensor thickness was 18.53 ± 3.16 nm when estimated by the viscoelastic model with four overtones (n= 3, 5, 7 and 9). The functionalized sensor exhibited a concentration-dependent frequency and dissipation shift upon interaction with empty MC3-LNPs of three different concentrations, which were then used to estimate the change in sensor thickness (Supplementary Figure 2a). The estimation shows that the average sensor thickness reached 58.37 ± 0.13 nm at 1 × 10^9^ particles/mL, which is close to the mean diameter of empty MC3-LNPs measured by DLS (56.80 ± 4.41 nm).

### 3.4 LNP–Protein Adsorption Kinetics

ApoE2 and ApoE3 are the alleles of the human ApoE gene differing only in one amino acid at residue 158 in the N-terminus (ApoE2: Cys158 and ApoE3: Arg158).^31,32^ Although the C-terminal domain of the two polymorphs preferentially binds to high-density lipoproteins (HDLs), the N-terminal domain of ApoE2 has a much lower binding affinity for low-density lipoprotein (LDL) receptors compared to ApoE3.^31^ Hence, we utilized the SPR method to investigate their binding kinetics toward LNPs, expecting they would display a similar trend in binding affinity since the putative lipid binding region in the C-terminus is identical. Serum albumin, the most abundant protein in the blood, is also known to be present in the LNP-protein corona.^33,34^ Therefore, we included it as another test protein of interest to examine the specificity and robustness of our assay (Supplementary Figure 3).

Figure 2c depicts the maximum binding signal (*R*_Max_) of the two ApoE alleles and HSA. Generally, *R*_Max_ of ∼100 RU was suitable to acquire sufficient resolution for kinetic assessment.^35,36^ The average *R*_Max_ of ApoE3 and ApoE2 was 125.3 ± 9.4 RU and 169.8 ± 3.6 RU, respectively, and they were much higher than that of HSA (13.5 ± 2.3 RU), highlighting the more favorable interaction between LNPs and ApoE proteins at the given concentration range (11–300 nM). The minimal interaction between HSA and our LNP formulations could be explained by the net surface charge of the LNPs. HSA tends to interact favorably with hydrophobic and positively charged nanoparticles.^37^ However, the two LNP formulations displayed close to neutral to slightly negative surface charge at pH 7.4, which is not optimal for HSA-LNP interaction. The acquired sensorgrams of the two ApoE polymorphs at 11–300 nM on the MC3-LNP-bound sensor depict the increase in relative response in accordance with the ApoE concentration with the detectable maximum response at 300 nM (Figure 2d). Under the same condition, increasing the ApoE concentration above 300 nM led to an inconsistent response trend due to the interference from the nonspecifically accumulated ApoE samples on the reference surface disrupting the active signals beyond the saturation point. Hence, we limited the protein concentration to 300 nM and below in our method for a reliable kinetic assessment.

In SPR, binding kinetics provide key implications in drug discovery by measuring the affinity of analyte-ligand interaction, which is useful for predicting the *in vivo* profile of drug candidates such as biodistribution, efficacy and side effects.^38,39^ In the 1:1 binding model, which assumes one analyte molecule binds to one ligand molecule, the association rate constant (*K*_a_) of ApoE3 was 1.71 × 10^5^ M^–1^ s^–1^, which was 2.5- and 7-fold higher than ApoE2 and HSA, respectively, as shown in Table 1a. All three proteins had minimal changes in the dissociation phase compared to the association phase (Figure 2d) and the dissociation rate constant (*K*_d_) of the three proteins was below the detection limit of the SPR instrument (<1.00 × 10^–5^ s^–1^), suggesting that the adsorbed proteins were tightly bound around the LNP surface to form a biomolecular corona. These findings were also confirmed by the QCM-D analysis (Supplementary Figure 2b), where the bound LNPs exhibited about a 3-fold higher frequency shift upon interacting with ApoE3 compared to that of HSA, even though the concentration of HSA (750 nM) was 2.5-fold higher than that of ApoE3 (300 nM) (Supplementary Figure 2c). The difference was even higher (40-fold) when ApoE3 was compared with BSA (750 nM).

**Table 1:** SPR Kinetic Assessment with the 1:1 Binding Model Showing the Association (*K*_a_) and Dissociation (*K*_d_) Rate of (a) Empty MC3-LNPs upon Interaction with HSA, ApoE3, and ApoE2 Proteins (11–300 nM) and (b) Comparison of the Average Association Rate between MC3- and SM102-LNPs with or without RNA upon Interaction with ApoE3 (11–300 nM) (*n* = 3)^a^

(a) MC3-LNP: Protein Interaction		
sample	*K*_a_ (M^–1^ s^–1^)	*K*_d_ (s^–1^)
HSA	2.44 × 10^4^	<1.00 × 10^–5^
ApoE3	1.72 × 10^5^	<1.00 × 10^–5^
ApoE2	6.73 × 10^4^	<1.00 × 10^–5^

(b) MC3 vs SM102 (Empty and RNA-Loaded): Interaction with ApoE3		
	*K*_a_ (M^–1^ s^–1^)	
sample	mean	SD
MC3 (Empty)^a^	1.59 × 10^5^	6.05 × 10^4^
MC3 (siRNA)^b^	4.29 × 10^4^	2.89 × 10^4^
SM102 (Empty)^b^	5.38 × 10^4^	3.71 × 10^3^
SM102 (mRNA)^b^	2.75 × 10^4^	2.32 × 10^4^

a Each letter next to the sample name denotes that the difference between groups is statistically significant (*p* < 0.05).

### 3.5 Binding Characteristics of MC3- and SM102-LNPs

MC3 and SM-102 are clinically approved and validated ionizable cationic lipids designed for delivering siRNA^9^ and mRNA,^16^ respectively. Figure 3a shows the sensorgrams and concentration–response plots of empty MC3-LNPs and SM102-LNPs, depicting their interaction with ApoE3 at various concentrations (11–300 nM). The response plots indicated that at the highest concentration point (300 nM), ApoE3 interacts with the detectable maximum response for the two at the same particle concentration (1 × 10^9^ particles/mL) and response level (∼1000 RU). Table 1b shows the average association rate constant of the two LNP formulations. In the 1:1 binding model, the association rate constant of empty MC3-LNPs (1.59 × 10^5^ M^–1^ s^–1^) was about 3-fold higher than that of SM102-LNPs (5.38 × 10^4^ M^–1^ s^–1^), suggesting more rapid interaction of ApoE3 with MC3-LNPs at this range of ApoE3 concentrations (*p* < 0.05). On the other hand, the average relative response of SM102-LNPs was slightly higher than that of MC3-LNPs at each ApoE concentration (Figure 3b). This is most likely due to the larger size of the SM102-LNPs (∼73 nm) than that of MC3-LNPs (∼60 nm) (Figure 1b). The size-dependent response was also observed between LNPs with or without RNAs of the two LNP formulations (Figure 3c). Here, the RNA-loaded LNPs exhibited slightly higher responses than those without RNAs and the mRNA-loaded SM102-LNPs achieved the highest average response as they have the largest particle size (∼102 nm). However, the differences among groups were not statistically significant. The size-dependent trend was also shown in the kinetics as the association rate constant of RNA-loaded LNPs was lower than those without RNAs, yet the difference was not significant except between MC3-LNPs with or without the siRNA cargoes (Table 1b). Since all LNP formulations had near-neutral surface charges at pH 7.4 (Figure 1c), the effect of surface charge was negligible when the study was performed in a running buffer (HBS, pH 7.4).

**Figure 3:**
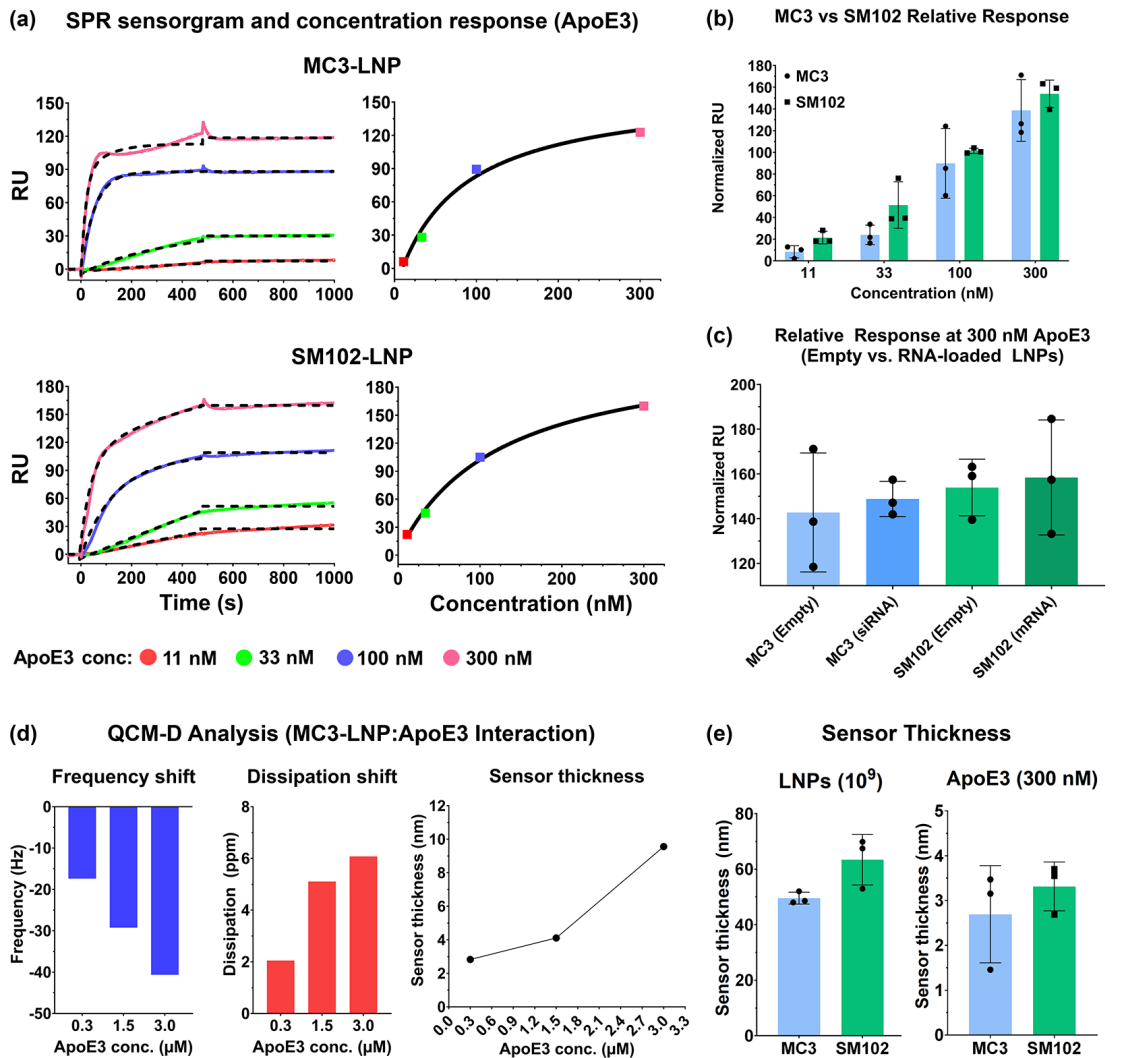
Binding assessment of MC3-LNPs and SM102-LNPs. (a) Representative SPR sensorgrams and concentration–response plots of empty MC3- and SM102-LNPs at various ApoE3 concentrations. (b, c) Normalized relative response plot of (b) empty MC3- and SM102-LNPs at 11–300 nM ApoE3 and (c) the two with or without the RNA cargoes at 300 nM ApoE3. (d) QCM-D analysis of MC3-LNPs depicting the shift in frequency, dissipation, and sensor thickness upon ApoE3 interaction of empty MC3-LNPs (1 × 10^9^ particles/mL). (e) Difference in the sensor thickness upon LNP association (1 × 10^9^ particles/mL) and LNP–ApoE3 interaction between MC3- and SM102-LNPs.

The QCM-D analysis (Figure 3d) also showed a concentration-dependent shift in frequency, dissipation, and sensor thickness at three ApoE3 concentrations (0.3, 1.5, and 3.0 μM). At equivalent particle concentration (1 × 10^9^ particles/mL), SM102-LNPs achieved a sensor thickness of 63.43 ± 9.14 nm, slightly higher than that of MC3-LNPs (56.80 ± 4.41 nm). Yet, there was no difference in ApoE binding between the two LNP formulations as indicated by the same level of sensor thickness (∼5.5 nm) achieved by the two at 300 nM ApoE3 (Figure 3e), which were comparable with the results from the SPR analysis (Figure 3b). Note that the QCM-D analysis used a much higher protein concentration (3.0 μM) than that of the SPR analysis (<300 nM). Furthermore, QCM-D required larger sample volumes (>1 mL) than SPR (100–200 μL) as the former has a much larger sensing area, leading to relatively less uniform measurement, a longer recording time and a higher risk of mass transport effect.^40^ Hence, we used solely the SPR workflow to investigate the species cross-reactivity in ApoE-LNP interactions.

### 3.6 Species Cross-Reactivity Assay

Establishing and understanding species cross-reactivity is an essential step for translational studies in drug discovery. Early in development, it is often necessary to evaluate new therapeutic modalities in relevant animal species, especially in the preclinical evaluation, including absorption, distribution, metabolism and excretion (ADME), efficacy, and toxicity studies.^41,42^ Non-human primate (NHP) models such as cynomolgus monkeys are commonly used in preclinical studies as they are genetically closer to humans than other animal species such as mice, rats, and dogs.^41^ Studies have shown that LNP delivery to NHP hepatocytes closely resembles those in humans.^43,44^ Recently, porcine models that share many physiological similarities with humans have also been widely used in biomedical research.^45,46^ Therefore, we employed our SPR workflow to investigate the interaction of empty MC3-LNPs with different ApoE homologs as a screening platform for species cross-reactivity.

Herein, the binding response and the kinetics of ApoE3 were compared with those of NHP (*cynomolgus macaques*), mouse, dog, and pig ApoE homologs prepared in varying concentrations (11–300 nM). Figure 4a shows the sensorgrams and response plots of the empty MC3-LNPs interacting with five ApoE homologs. Note that all samples reached the saturation point at 300 nM except for the pig and dog homologs, as depicted in their response plots. The linearly increasing curves suggest that pig and dog homologs have less affinity toward LNPs as they require higher concentrations or longer exposure time to reach saturation. The kinetic assessment with the 1:1 binding model in Table 2a shows that the NHP and mouse homologs displayed about the same level of association as ApoE3. Conversely, the association rate constants of the pig and dog homologs were much lower than the rest, which aligns with the results from the response plot (Figure 4a). The normalized responses of the ApoE homologs at various concentrations indicate that the response level of the dog ApoE was consistently lower than the rest (Figure 4b). The phylogenetic tree and the similarity matrix in Figure 4c depict the primary sequence similarity of ApoE3 with the four ApoE homologs. The pig and dog ApoE, having the least similarity (<70%) with ApoE3, had the lowest association rates, whereas NHP ApoE, having the most similarity (93.38%), closely resembled the kinetic behavior of ApoE3.

**Figure 4:**
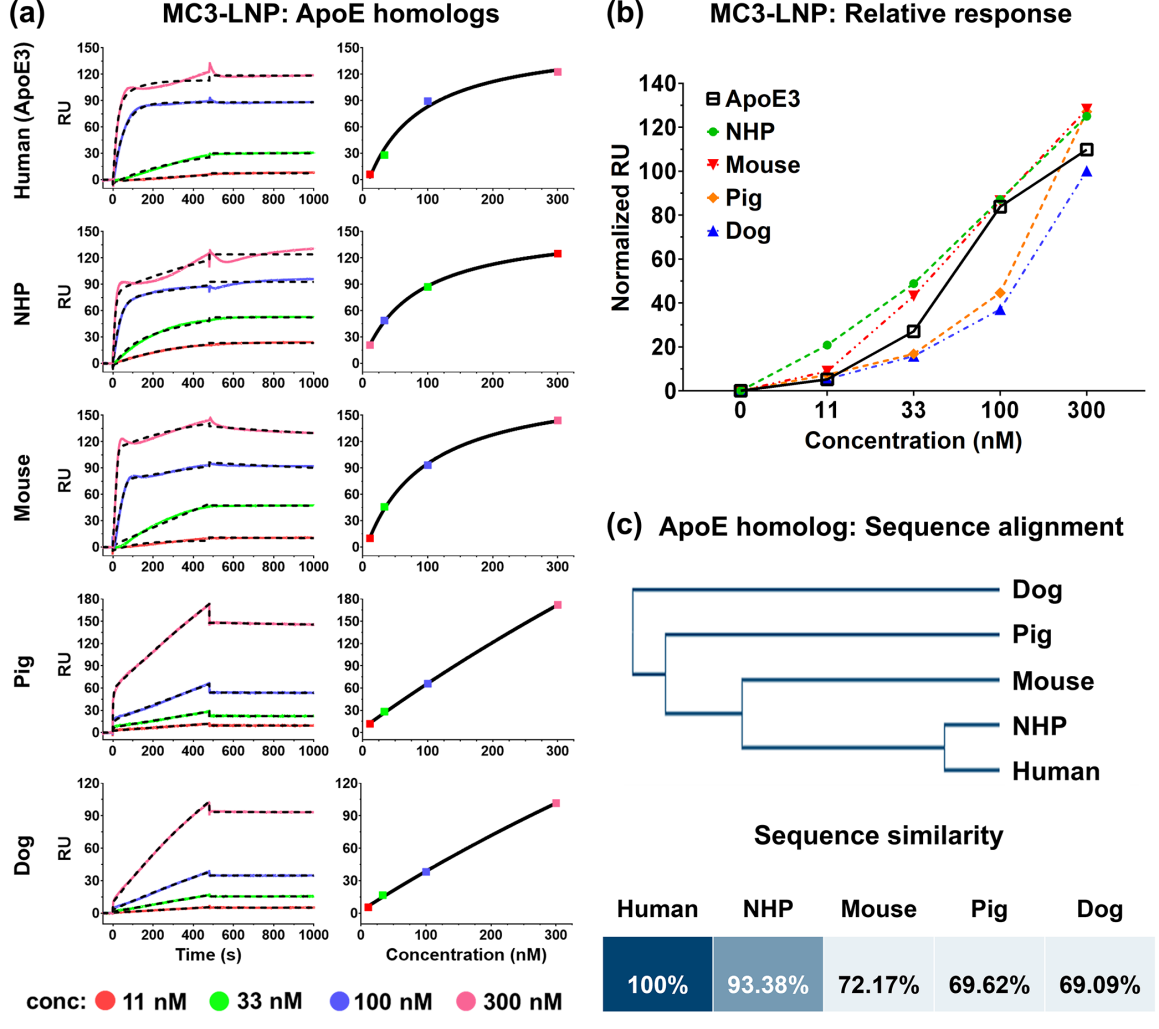
Species cross-reactivity assessment. (a) Representative sensorgrams and concentration–response plots of empty MC3-LNPs interacting with five ApoE homologs. (b) Normalized relative responses of the five homologs at various concentrations. (c) Phylogenetic tree and cross-species similarity matrix depicting the sequence similarity among human, NHP, mouse, pig, and dog ApoE homologs.

**Table 2:** SPR Kinetic Assessment Showing the Association (*K*_a_) Rate of Empty MC3-LNPs Interacting with Five ApoE Homologs (11–300 nM) Estimated by (a) the 1:1 Binding Model and (b) the Two-Step Binding Model

	binding model		
	(a) 1:1 binding (A + B ⇄ AB)	(b) two-step binding (A + B ⇄ AB ⇄ AB_*x*_)	
sample	*K*_a_ (M^–1^ s^–1^)	*K*_a1_ (M^–1^ s^–1^)	*K*_a2_ (M^–1^ s^–1^)
ApoE3	1.72 × 10^5^	3.20 × 10^5^	7.16 × 10^–3^
ApoE-NHP	1.27 × 10^5^	1.29 × 10^5^	1.35 × 10^–2^
ApoE-Mouse	1.87 × 10^5^	1.54 × 10^9^	5.61 × 10^–3^
ApoE-Pig	4.25 × 10^3^	5.45 × 10^3^	8.32 × 10^–3^
ApoE-Dog	7.52 × 10^3^	9.03 × 10^3^	2.54 × 10^–4^

Numerous factors other than amino acid sequence could also contribute to the binding interaction between ApoE and LNPs. For instance, it is proposed that ApoE3 sequentially follows a two-step process of conformation change to form a stable complex with lipid particles.^47^ Essentially, it requires engagement of both the C-terminal and N-terminal domains, the former acting more rapidly than the latter. On the contrary, mouse ApoE behaves more like a single folded domain to form a complex in a single-step manner.^48^ Moreover, it has a higher affinity toward very low-density lipoproteins (VLDLs) than HDLs, which ApoE3 favors. To better understand the kinetics in the perspective of two-step binding conformation, the two-step binding model was applied to the SPR sensorgrams of the five ApoE homologs to assess the association kinetics following the previously reported method.^47,49^ The first and second steps of the two-step binding model could represent the binding kinetics of the C-terminal and N-terminal domains of the ApoE protein, respectively. Here, the association rate constant of the first step (*K*_a1_) was about the same as that of the 1:1 binding model for all ApoE homologs except for the mouse ApoE (Table 2b). It shows that mouse ApoE has a much higher initial association rate (*K*_a1_ = 1.54 × 10^9^ M^–1^ s^–1^) than the others, which captures the one-domain binding behavior of the mouse ApoE more effectively than the 1:1 binding model (*K*_a_ = 1.87 × 10^5^ M^–1^ s^–1^). The second association kinetic constant (*K*_a2_) of the five homologs exhibited a much slower binding kinetic than *K*_a1_ and since the values are below the assessment threshold of the SPR instrument (10^3^ M^–1^ s^–1^), we could infer that there is only a trivial kinetic impact of the N-terminal domain in ApoE-LNP interaction.

This is meaningful as these findings demonstrate the feasibility of implementing the SPR routine as an early stage screening tool for LNP drug development. Our assay clearly demonstrated that even little changes in the sequence, conformation, and size could influence the overall stability of the lipid binding behavior of different ApoE homologs.^47,50,51^ In this work, we only focused on two LNP formulations and their interactions with ApoE, as it is the key contributor to cellular uptake. The method could be extended and utilized as a high throughput screening platform by employing different binding models to characterize the interaction of various target-specific LNP formulations (e.g., difference in size, surface charge and lipid composition) with specific proteins or receptors (e.g., serum albumin and fibrinogen).

## 4 Conclusions

Our rapid and robust SPR method orthogonal to the conventional QCM-D method could provide new insight into the mechanism of LNP interaction with different target proteins. Comparing the association of each protein candidate at low concentrations allows us to understand how rapidly and tightly the protein corona forms around the LNP surface. Additionally, we demonstrated how different ApoE homologs interact with the LNP surface through species cross-reactivity assay with two binding models. This can aid the selection of appropriate animal model(s) suitable for *in vivo* investigations of the LNP modality as a drug product. This SPR LNP binding workflow will enhance the selection of optimal LNP formulations for targeted biodistribution to a variety of target organs or tissues.

## Supplementary Material



## Notes

Flow nanoanalyzer scatter plots of empty and RNA-loaded LNPs, QCM-D analysis showing the change in frequency, dissipation and sensor thickness upon association of LNPs on the functionalized sensor and corresponding interaction with different protein types and concentrations, SPR sensorgram and concentration response plot depicting the interaction between LNPs and HSA (PDF)

## ∇

Development Science, AbbVie, Irvine, CA 92612, USA

## Abbreviations

ADME: Absorption, distribution metabolism and excretion
aPEG: Biotin anti-PEG antibody
ApoE: Apolipoprotein E
BSA: Bovine serum albumin
DLS: Dynamic light scattering
HBS: HEPES-buffer saline
HDL: High-density lipoprotein
HSA: Human serum albumin
LDLR: Low-density lipoprotein receptor
LNP: Lipid nanoparticle
MC3: D-Lin-MC3-DMA
NTA: Nanoparticle tracking analysis
N/P: Amine group in the ionizable lipid/phosphate group in the RNA cargo
NHP: Non-human primate
PEG: Polyethylene glycol
QCM-D: Quartz crystal microbalance with dissipation
SPR: Surface plasmon resonance
VLDL: Very low-density lipoprotein
1,2-DSPC: 1,2-Distearoyl-*sn*-glycero-3-PC
