# Supplementary material for: Surface Plasmon Resonance Based Binding Characterization for Screening RNA-Loaded Lipid Nanoparticles (LNPs): Exploring Species Cross-Reactivity in LNP–Apolipoprotein E Interactions
Source: Mol Pharm. 2025 Jul 7;22(8):4587–96. doi: 10.1021/acs.molpharmaceut.5c00068 (PMC12326350; doi:10.1021/acs.molpharmaceut.5c00068)
Supplement: Supplementary file 1 [file mp5c00068_si_001.pdf]

Surface Plasmon Resonance Based Binding Characterization for Screening RNA-Loaded Lipid Nanoparticles (LNPs): Exploring Species Cross-Reactivity in LNP-Apolipoprotein E Interactions

Benjamin Lew[†,‡], Sandeep Chhabra[†], Jacob A. Lewis[†], Angela C. Wagoner[†], Angel Hsu[†], Steve Halaby[†,∇], Pooja Sharma[§], Justin K. Murray[‖], Francis Kinderman[†], Deirdre Murphy Piedmonte[†], and Brendan R. Amer[†,*]

[†]Pre-Pivotal Drug Product Technologies, Process Development, Operations, Amgen, Thousand Oaks, California, 91320, United States

[‡]Amgen Postdoctoral Fellow Program, Amgen, Thousand Oaks, California, 91320, United States

[§]Lead Discovery and Characterization, Research, Amgen, Thousand Oaks, California, 91320, United States

[‖]Complex Biologics, Amgen Research, Amgen, Thousand Oaks, California, 91320, United States

[*]Email: bamer@amgen.com

[∇] Current affiliation: Development Science, AbbVie, Irvine, CA, USA 92612

SUPPLEMENTARY FIGURES

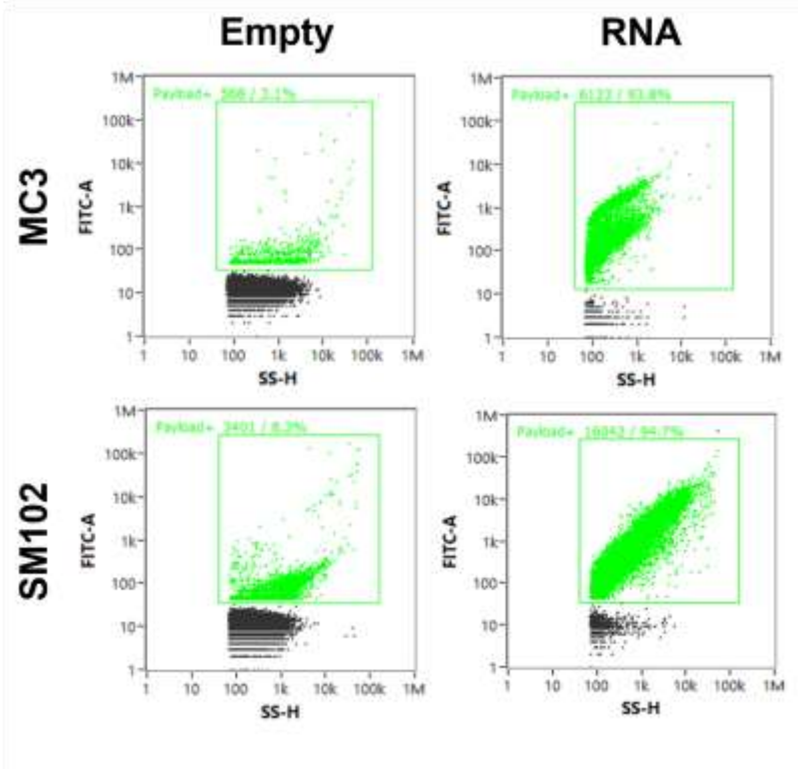

Supplementary Figure 1. RNA loading efficiency assessment. Analysis of RNA-LNPs by labeling with nucleic acid stain showing the distribution of siRNA and mRNA cargoes (green box) within or outside the MC3- and SM102-LNPs, respectively. The empty LNPs were used as a control.

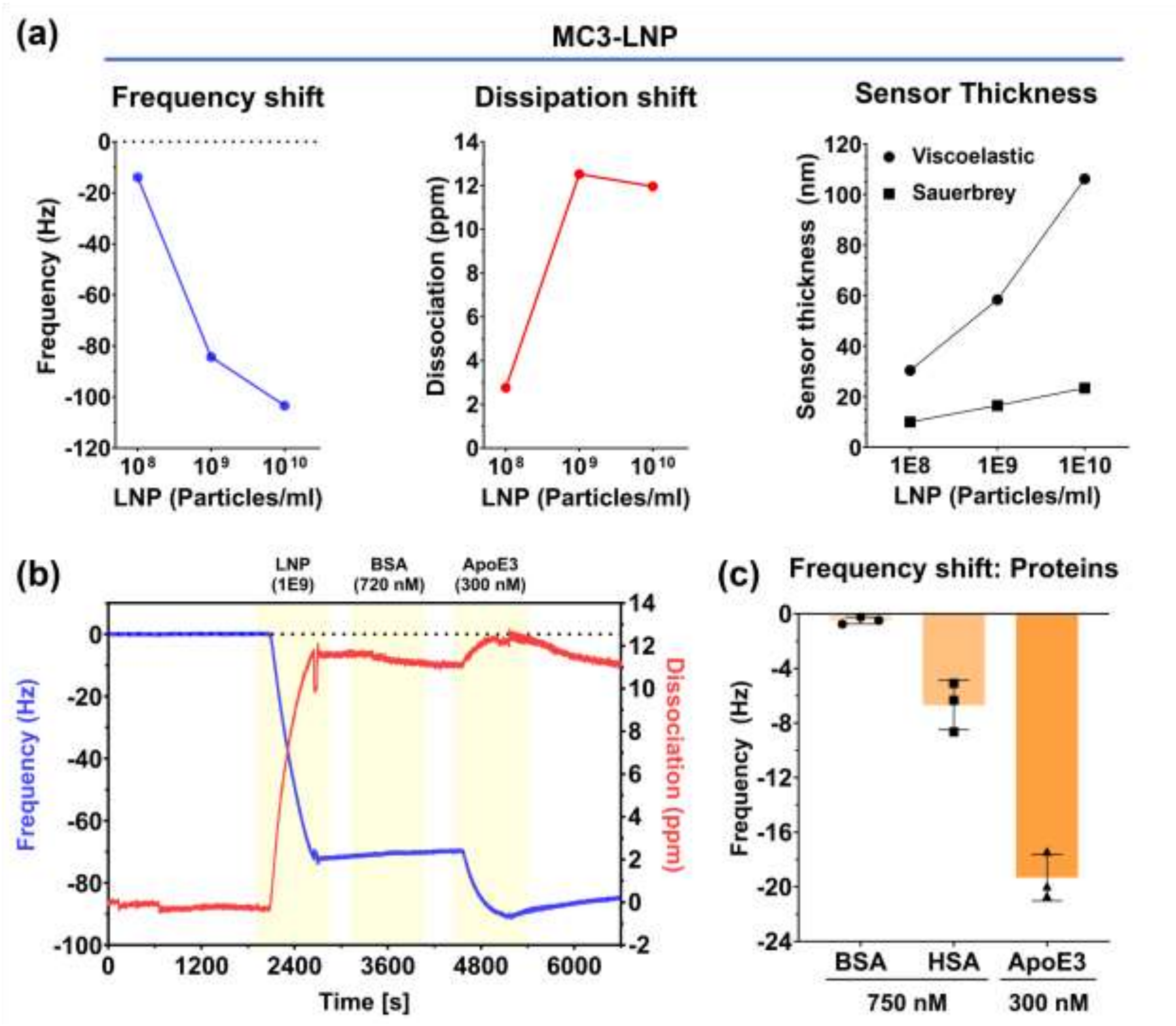

Supplementary Figure 2. QCM-D analysis. (a) Change in frequency, dissipation, and sensor thickness upon association of empty MC3-LNPs of three different concentrations. The sensor thickness was assessed by viscoelastic and Sauerbrey models. (b) Representative QCM-D signal plot depicting the shift in frequency and dissipation in accordance with the LNP association and LNP-protein interactions. (c) Frequency shift of the LNP immobilized sensor (MC3-LNPs, 1×10^9 particles/mL) upon interaction with BSA (750 nM), HSA (750 nM), and ApoE3 (300 nM).

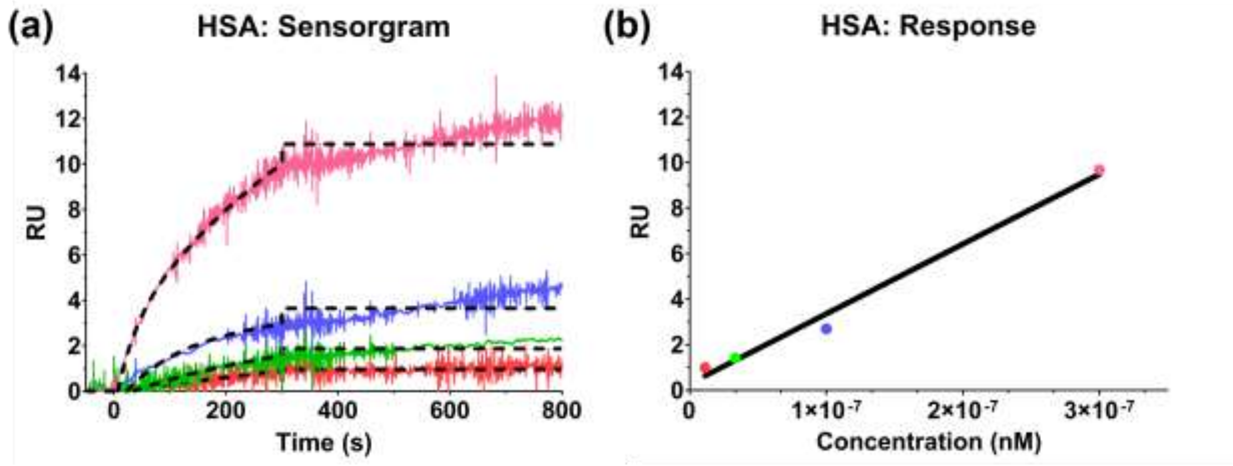

Supplementary Figure 3. MC3-LNP HSA interaction. (a) Representative sensorgram and (b) concentration-response of empty MC3-LNPs upon interacting with HSA at varying concentrations (11-300 nM).
